# Efficient Equality Test on Identity-Based Ciphertexts Supporting Flexible Authorization

**DOI:** 10.3390/e25020362

**Published:** 2023-02-15

**Authors:** Na Li

**Affiliations:** State Key Laboratory of Networking and Switching Technology, Beijing University of Posts and Telecommunications, Beijing 100876, China; lina_2014@163.com

**Keywords:** equality test, trapdoor discrete log groups, identity-based encryption, cloud storage

## Abstract

In the cloud, uploading encrypted data is the most effective way to ensure that the data are not leaked. However, data access control is still an open problem in cloud storage systems. To provide an authorization mechanism to limit the comparison of a user’s ciphertexts with those of another, public key encryption supporting the equality test with four flexible authorizations (PKEET-FA) is presented. Subsequently, more functional identity-based encryption supporting the equality test (IBEET-FA) further combines identity-based encryption with flexible authorization. The bilinear pairing has always been intended to be replaced due to the high computational cost. Hence, in this paper, we use general trapdoor discrete log groups to construct a new and secure IBEET-FA scheme, which is more efficient. The computational cost for the encryption algorithm in our scheme was reduced to 43% of that of the scheme of Li et al. In Type 2 and 3 authorization algorithms, the computational cost of both was reduced to 40% of that of the scheme of Li et al. Furthermore, we give proof that our scheme is secure against one-wayness under the chosen identity and chosen ciphertext attacks (OW-ID-CCA), and indistinguishable against chosen identity and chosen ciphertext attacks (IND-ID-CCA).

## 1. Introduction

With the application of the Internet increasingly spreading, people have more extensive storage and computing requirements for cloud servers. Users make full use of cloud servers, allowing cloud servers to help them in storing and processing data, reducing the user’s storage burden and computing overhead. Users in different regions can upload data onto and download data from a server, which provides convenience for users to share data. However, servers are also vulnerable to some attacks. If users store their data unencrypted in the cloud server, attackers or malicious internal administrators may access the data stored by users. The solution is for every user to upload encrypted data onto the cloud server. Previous classical encryption schemes cannot realize direct searches or calculations in the ciphertext.In a searchable encryption scheme [1], the ciphertext and trapdoor for retrieval need to be obtained with the same public and private key pair.

A novel PKEET scheme [2] was first proposed by Yang et al. in 2010. In this scheme, users can test whether ciphertexts encrypted by different public keys contain the same plaintext without decrypting the ciphertext, which avoids the previous limitations of searchable encryption. However, in the scheme, anyone can test the encrypted data, which can lead to data leakage. Taking into account better meeting practical applications, Tang proposed a fine-grained equality test scheme [3] that can achieve fine-grained authorization by sending tokens to a proxy. The equality test of flexible authorization for more scenarios was proposed in [4], in which there were different authorizations to meet the different needs of users, and different authorization types corresponded to different test permissions. It can not only perform the equivalence testing of ciphertext that was encrypted without the same public key, but also designate testers, which better protects the privacy of the data. On this basis, to avoid the public key infrastructure (PKI), a functional and efficient IBEET-FA scheme [5] is proposed as a new concept, replacing PKE with IBE. The first IBE scheme [6] replaced the public key with user-related identity information, and the private key is calculated and provided by a trusted third party. No need for a public key means that the difficulty of key management is eliminated. A new IBE scheme [7] using the general trapdoor discrete logarithm group was proposed that reduces the computational cost compared to that when using bilinear pairs. IBEET-FA [5] is based on bilinear pairing.

### 1.1. Our Contribution

Bilinear pairing is computationally expensive, and to reduce the computational cost, we have attempted to replace pairing with discrete logarithms. We reconstructed an existing concept with a different tool, namely, reconstructing the IBEET-FA scheme with discrete logarithms. This can achieve more efficient searches in ciphertexts encrypted by different public keys, and maintain the nature of flexible authorization in which different authorizations correspond to different permissions. A public key infrastructure is not required.

We first defined the scheme and its correctness. Subsequently, a specific scheme IBEET-FA without paring was constructed, and the scheme was proven to be correct. Our scheme is communicationally efficient, and it has a small public key and ciphertext. The scheme is computationally efficient, as the Aut-1, Aut-2, and Aut-3 authorization algorithms and testing algorithms in it all have a small computational overhead.

We then define two security models for the scheme, and two types of adversaries, Adv-I and Adv-II. Our IBEET-FA without a pairing scheme achieved OW-ID-CCA security for Aut-γ (γ = 1, 2, 3) against Adv-I on the basis of the CDH assumption in the random oracle model. The IBEET-FA without a pairing scheme achieved IND-ID-CCA security for Aut-γ (γ = 1, 2, 3) against Adv-II on the basis of the DDH assumption.

### 1.2. Related Works

A new concept of public key encryption with keyword search (PEKS) was proposed by Boneh et al. [1] in 2004 that allows for direct keyword searches in ciphertext without decrypting the ciphertext. A user can generate the corresponding trapdoor of some keyword by using its private key and perform a keyword search in the ciphertexts with the trapdoor. Subsequently, many related variants were proposed [8,9,10]. Bellare et al. [11] proposed a deterministic PKE scheme. Yang et al. [2] devised a ciphertext-based equality test scheme using bilinear groups for searchable and classified encrypted data. However, in that scheme, anyone could perform the test, so it is easy for it to cause data leakage, which is not conducive to data privacy. Tang [3] presented a new method where two users could authorize a proxy to execute equality calculation on their encrypted message by issuing tokens. Tang [12] gave a new PKE in a two-proxy model supporting fine-grained authorization (FG-PKEET) in which the two proxies were required to cooperate to complete the equality test. Subsequently, Tang [13] proposed the construction of an all-or-nothing PKEET (AoN-PKEET).

A new scheme of PKE with a delegated equality test (PKE-DET) was proposed by Ma et al. in [14]; in a multiuser model, only the delegated party can perform the equality test. Wu et al. [15] introduced a new equality test concept that could achieve security against insider attacks. Ma [16] proposed a variant of PKEET in which a cloud server could directly execute the equality test on the ciphertexts of the specified user, realizing the security of the cloud database application. In [17], PKE-AET offered a new idea regarding two different kinds of warrants, namely, receiver warrants and cipher warrants. After a tester receives a receiver warrant from some receiver, the tester can perform the equality test on any of the receiver’s ciphertext; in the second case, after a tester receives a cipher warrant associated with some ciphertext from some receiver, the tester can just execute an equality test on that ciphertext. Huang et al. [18] presented a ciphertext-binded authority (CBA) PKEET scheme. CBAs are only valid for specific ciphertexts, and they are invalid for other ciphertexts encrypted by the same public key. The concept of the filtered equality test (FET) was proposed by Huang et al. [19] where the receiver selects a set of messages and generates the corresponding warrant. After a user receives the warrant, if the plaintext corresponding to the ciphertext is in the message set, they can perform an equality test on the recipient’s ciphertext. Huang et al. [20] proposed a PKE-FET scheme in which FET was also applied to construct searchable encryption. The key policy-attribute-based encryption with an equality test scheme was proposed by Zhu et al. in [21]. After the flexible scheme, a ciphertext policy-attribute-based encryption scheme was presented by Wang et al. [22] that also supported the function of the equality test.

A new authorization mechanism for efficient PKEET-FA was proposed by Ma et al. [4], which can more effectively achieve user privacy protection. The scheme was based on bilinear pairing, Lin et al. [23] made improvements on this basis and proposed a novel PKEET-FA scheme, Bilinear pairings were not used in this scheme. This protocol used a quadratic curve to do the equality test, Zhu et al. [24] used a simpler straight line for the equality test. A new concept of IBEET by combining two existing concepts PKEET and IBE was given by Ma et al. [25]. A new IBEET-FA scheme was proposed in [5]. Users can directly execute equality tests on the ciphertext, eliminating the need for complex key management.

Duong et al. [26] proposed a new PKEET scheme based on ideal lattices and a scheme based on integer lattices, both schemes can achieve CCA2-security. Ref. [27] introduced the trends in multimedia forensics, and many deep-learning-based techniques. In [28], lSusilo et al. presented a novel concept of public key encryption with multi-ciphertext equality test (PKE-MET), which enables the cloud server to perform equality tests among multiple ciphertexts. A new primitive of identity-based encryption with equality test and datestamp-based authorization mechanism (IBEET-DBA) was proposed by Lin et al. [29], in which the data owner could control the valid period of trapdoor by using datestamp. Deverajan et al. [30] presented public key encryption with equality test based on discrete logarithm problem (DLP). Considering the possible attacks on trapdoors given to cloud servers and the different computing power of the entities, Vaanchig et al. [31] introduced a notion of secure-channel-free IBEET (SCF-IBEET).

### 1.3. Organization

We organize the remainder of the paper as follows. The definitions of Trapdoor Discrete Log Groups and Decision Diffie–Hellman Problem are given in Section 2. Then, we give the system model, the definitions of IBEET-FA and the security model in Section 3. In Section 4, we propose a new IBEET-FA scheme without pairing. In Section 5, the security analysis of our scheme will be given. In Section 6, we will show the complexity comparison of our scheme and other related schemes. In the last section, some conclusions will be given.

## 2. Preliminaries

### 2.1. Trapdoor Discrete Log (TDL) Groups

**Definition** **1.** *A TDL group generator consists of algorithms TDLGen and SolveDL:*

- *TDLGen(k): Given security parameter k as the input, the algorithm returns a tuple (T,q,g,G) where T is used to denote the trapdoor, q is used to denote the prime order, g is used to denote a random generator, and G is used to denote a group.*
- *SolveDL(k,(T,q,g,G),h): Given the inputs of a security parameter k, (T,q,g,G) denoting a tuple and h denoting a group element, the algorithm outputs $\alpha \in Z_{q}$, and $h=g^{\alpha }$ holds.*

### 2.2. Computational Diffie–Hellman (CDH) Problem

**Definition** **2.** *Let q be the prime order of group G, generator g is gotten from the running result of algorithm TDLGen in the Definition1, let $\left(g,g^{a},g^{b}\right)$ be a tuple in G, for $a,b\in Z_{q}$. It is intractable to compute $g^{ab}$. A is an adversary, in probability polynomial time, the advantage of adversary A to solve the CDH problem is*

$$\begin{array}{c} Adv_{A,G}^{CDH}\left(k\right)=P\left(A\left(g,g^{a},g^{b}\right)=g^{ab},G\right) \end{array}$$

### 2.3. Decision Diffie–Hellman (DDH) Problem

**Definition** **3.** *Let q be the prime order of group G, generator g is gotten from the running result of algorithm TDLGen in the Definition1, let $\left(g,g^{a},g^{b},g^{c}\right)$, $\left(g,g^{a},g^{b},g^{ab}\right)$ be two tuples in G, for $a,b,c\in Z_{q}$. It is difficult to distinguish the two tuples in this computational relationship. A is an adversary, in probability polynomial time, the advantage of A to solve the DDH problem is*

$$\begin{array}{c} Adv_{A,G}^{DDH}\left(k\right)=\left|P\left(A\left(g,g^{a},g^{b},g^{ab}\right)=1,G\right)-P\left(A\left(g,g^{a},g^{b},g^{c}\right)=1,G\right)\right| \end{array}$$

## 3. System Model and Definition

In Section 3.1 and Section 3.2, we give the system model and the definition of IBEET-FA, similarly in [5]. In Section 3.3, we give the security model of IBEET-FA.

### 3.1. System Model

**Figure 1:**
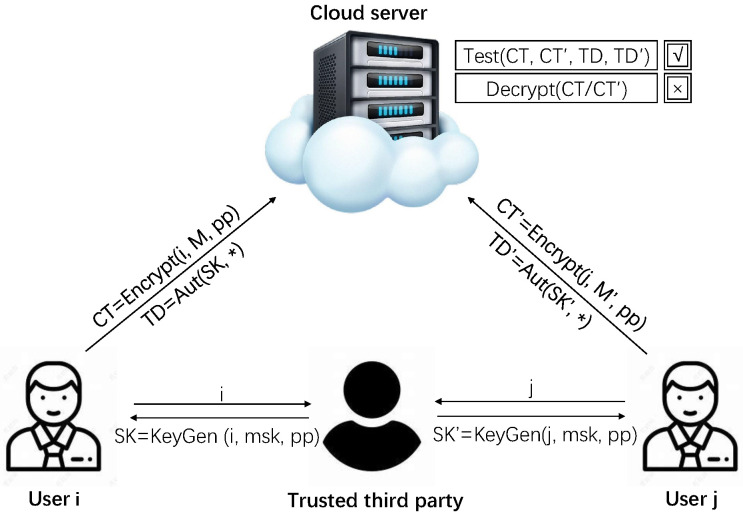
IBEET-FA system model.

In our defined IBEET-FA scheme, we give four entities: a cloud server, a trusted third party, and two users labeled as *i* and *j*. The trusted third party generates system parameters for users and cloud service. User *i* and user *j* encrypt their data with their public key, and store ciphertext in the cloud server, and the cloud server is authorized to do equality tests on stored ciphertext, but the server does not have the ability to decrypt them. We present the IBEET-FA system model in Figure 1.

### 3.2. Definition of IBEET-FA

**Definition** **4.** *Our IBEET-FA scheme consists of four algorithms:*

- *Setup(k): Taken security parameter k as the input, the public parameter pp and the master secret key msk will be gotten from the running result of the algorithm.*
- *KeyGen(i,msk,pp): Given label i, master secret key msk, and public parameter pp as input, the algorithm returns the secret key $SK=(\alpha_{i},\beta_{i})$.*
- *Encrypt(i,M,pp): Given the inputs of user i, a message M and public parameter pp, the algorithm returns the ciphertext CT.*
- *$Decrypt(i,\alpha_{i},CT,pp)$: Given label i, a private key $\alpha_{i}$, a ciphertext CT and public parameter pp as inputs, a message M will be gotten from the running result of the algorithm, or returns an error symbol* ⊥.

User *i* has the public-secret key pair (i,SK), corresponding encrypted data is CT, User *j* has the public-secret key pair $\left(j,SK^{{}^{\prime }}\right)$, corresponding encrypted data is $CT^{{}^{\prime }}$. They have four types of authorization, corresponding to four different Aut algorithms and four different Test algorithms. Aut algorithm is used to generate trapdoors for users, and the cloud service runs Test procedure to test whether or not two different encrypted data contain the same message.

Aut-1:

- $Aut_{1}\left(i,SK\right)$: Given user *i* and *i*’s secret key SK as inputs, the authorization procedure returns a trapdoor $TD_{1}$.
- $Test_{1}\left(CT,CT^{{}^{\prime }},TD_{1},TD_{1}^{{}^{\prime }}\right)$: Given the inputs of *i*’ciphertext CT, *i*’trapdoor $TD_{1}$, *j*’ciphertext $CT^{{}^{\prime }}$ and *j*’trapdoor $TD_{1}^{{}^{\prime }}$, the test procedure returns 1 if two ciphertexts contain the same message, otherwise returns 0.

Aut-2:

- $Aut_{2}\left(SK,CT\right)$: Given the inputs of user *i*’private key SK and a ciphertext CT, the authorization procedure outputs a trapdoor $TD_{2}$.
- $Test_{2}\left(CT,CT^{{}^{\prime }},TD_{2},TD_{2}^{{}^{\prime }}\right)$: Given the inputs of *i*’ciphertext CT, *i*’trapdoor $TD_{2}$, *j*’ciphertext $CT^{{}^{\prime }}$ and *j*’trapdoor $TD_{2}^{{}^{\prime }}$, the test procedure returns 1 if two ciphertexts contain the same plaintext, otherwise returns 0.

Aut-3:

- $Aut_{3}\left(SK,CT,CT^{{}^{\prime }}\right)$: Given the inputs of user *i*’private key SK, *i*’ciphertext CT, and *j*’ciphertext $CT^{{}^{\prime }}$, the authorization procedure outputs a trapdoor $TD_{3}$.
- $Test_{3}\left(CT,CT^{{}^{\prime }},TD_{3},TD_{3}^{{}^{\prime }}\right)$: Given the inputs of *i*’ciphertext CT, *i*’trapdoor $TD_{3}$, *j*’ciphertext $CT^{{}^{\prime }}$ and *j*’trapdoor $TD_{3}^{{}^{\prime }}$, the test procedure returns 1 if two ciphertexts contain the same plaintext, otherwise returns 0.

Aut-4:

- $Aut_{4}\left(SK,CT\right)$: Given the inputs of user *i*’private key SK and ciphertext CT, the authorization procedure returns a trapdoor $TD_{4}$.
- $Aut_{4}\left(j,SK^{{}^{\prime }}\right)$: Given user *j* and *j*’s secret key $SK^{{}^{\prime }}$ as inputs, the authorization procedure returns a trapdoor $TD_{4}^{{}^{\prime }}$.
- $Test_{4}\left(CT,CT^{{}^{\prime }},TD_{4},TD_{4}^{{}^{\prime }}\right)$: Given the inputs of *i*’ciphertext CT, *i*’trapdoor $TD_{4}$, *j*’ciphertext $CT^{{}^{\prime }}$ and *j*’trapdoor $TD_{4}^{{}^{\prime }}$, the test procedure returns 1 if two ciphertexts contain the same message, otherwise returns 0.

**Definition** **5.** *(Correctness): If for any (msk,pp)←Setup(k), $\left(\alpha_{i},\beta_{i}\right)\leftarrow KeyGen\left(msk,pp,i\right)$, $\left(\alpha_{j},\beta_{j}\right)\leftarrow KeyGen\left(msk,pp,j\right)$, the following conditions can be satisfied, we say an IBEET-FA scheme is correct.*

1. For any possible plaintext *M* in the plaintext space, $Decrypt(Encrypt\left(M,i,pp\right),pp,\alpha_{i},i)=M$, all equations hold.
2. For any possible ciphertext CT of user *i* and any possible ciphertext $CT^{{}^{\prime }}$ of user *j*, if Decrypt$\left(i,\alpha_{i},CT,pp\right)=Decrypt\left(j,\alpha_{j},CT^{{}^{\prime }},pp\right)\neq ⊥$:
  Aut-1: For two trapdoors of $Aut_{1}\left(i,SK\right)=TD_{1}$, $Aut_{1}\left(j,SK^{{}^{\prime }}\right)=TD_{1}^{{}^{\prime }}$, the following equality always holds that
  $$\begin{array}{c} Test_{1}\left(CT,TD_{1},CT^{{}^{\prime }},TD_{1}^{{}^{\prime }}\right)=1. \end{array}$$
  Aut-2: For two trapdoors of $Aut_{2}\left(SK,CT\right)=TD_{2}$, $Aut_{2}\left(SK^{{}^{\prime }},CT^{{}^{\prime }}\right)=TD_{2}^{{}^{\prime }}$, the following equality always holds that
  $$\begin{array}{c} Test_{2}\left(CT,TD_{2},CT^{{}^{\prime }},TD_{2}^{{}^{\prime }}\right)=1. \end{array}$$
  Aut-3: For two trapdoors of $Aut_{3}\left(SK,CT,CT^{{}^{\prime }}\right)=TD_{3}$, $Aut_{3}\left(SK^{{}^{\prime }},CT^{{}^{\prime }},CT\right)=TD_{3}^{{}^{\prime }}$, the following equality always holds that
  $$\begin{array}{c} Test_{3}\left(CT,TD_{3},CT^{{}^{\prime }},TD_{3}^{{}^{\prime }}\right)=1. \end{array}$$
  Aut-4: For two trapdoors of $Aut_{4}\left(SK,CT\right)=TD_{4}$, $Aut_{4}\left(j,SK^{{}^{\prime }}\right)=TD_{4}^{{}^{\prime }}$, the following equality always holds that
  $$\begin{array}{c} Test_{4}\left(CT,TD_{4},CT^{{}^{\prime }},TD_{4}^{{}^{\prime }}\right)=1. \end{array}$$
3. For any possible ciphertext CT of user *i* and any possible ciphertext $CT^{{}^{\prime }}$ of user *j*, if Decrypt$\left(i,\alpha_{i},CT,mpk\right)\neq Decrypt\left(j,\alpha_{j},CT^{{}^{\prime }},mpk\right)$, where ϵ(·) be a negligible function about *k*:
  Aut-1: For two trapdoors of $Aut_{1}\left(i,SK\right)=TD_{1}$, $Aut_{1}\left(j,SK^{{}^{\prime }}\right)=TD_{1}^{{}^{\prime }}$, the following equality always holds that
  $$\begin{array}{c} P\left[Test_{1}\left(CT,TD_{1},CT^{{}^{\prime }},TD_{1}^{{}^{\prime }}\right)=1\right]\leq \epsilon \left(k\right). \end{array}$$
  Aut-2: For two trapdoors of $Aut_{2}\left(SK,CT\right)=TD_{2}$, $Aut_{2}\left(SK^{{}^{\prime }},CT^{{}^{\prime }}\right)=TD_{2}^{{}^{\prime }}$, the following equality always holds that
  $$\begin{array}{c} P\left[Test_{2}\left(CT,TD_{2},CT^{{}^{\prime }},TD_{2}^{{}^{\prime }}\right)=1\right]\leq \epsilon \left(k\right). \end{array}$$
  Aut-3: For two trapdoors of $Aut_{3}\left(SK,CT,CT^{{}^{\prime }}\right)=TD_{3}$, $Aut_{3}\left(SK^{{}^{\prime }},CT^{{}^{\prime }},CT\right)=TD_{3}^{{}^{\prime }}$, the following equality always holds that
  $$\begin{array}{c} P\left[Test_{3}\left(CT,TD_{3},CT^{{}^{\prime }},TD_{3}^{{}^{\prime }}\right)=1\right]\leq \epsilon \left(k\right). \end{array}$$
  Aut-4: For two trapdoors of $Aut_{4}\left(SK,CT\right)=TD_{4}$, $Aut_{4}\left(j,SK^{{}^{\prime }}\right)=TD_{4}^{{}^{\prime }}$, the following equality always holds that
  $$\begin{array}{c} P\left[Test_{4}\left(CT,TD_{4},CT^{{}^{\prime }},TD_{4}^{{}^{\prime }}\right)=1\right]\leq \epsilon \left(k\right). \end{array}$$

### 3.3. Security Model

According to the nature of our scheme, we use the IBEET-FA security models defined in [5]. Since Aut-4 is a combination of one user authorization way in Aut-1 and one user authorization way in Aut-2, we omit Aut-4 authorization queries for simplicity. Adversaries are only allowed to query for Aut-γ (γ = 1, 2, 3). We define two kinds of adversaries for the security model of our IBEET-FA scheme:

- Adv-I: For Aut-γ (γ = 1, 2, 3), with Aut-γ trapdoor information, the adversary can not get the plaintext from the challenge ciphertext.
- Adv-II: For Aut-γ (γ = 1, 2, 3), without Aut-γ trapdoor information, the adversary can not know the challenge ciphertext is from which plaintext.

Under chosen ciphertext and chosen identity attacks, We now define the one-wayness security (OW-ID-CCA) against Adv-I for Aut-γ (γ = 1, 2, 3) as follows:

GameI: Let the receiver have index *t* (1≤t≤n), and assume $A_{1}$ is a Adv-I. Between the challenger $C_{1}$ and the adversary $A_{1}$, the game goes as follows:

- Setup: Challenger $C_{1}$ firstly picks *k* as a security parameter, then gets public parameter pp by calling Setup algorithm, sends pp to $A_{1}$.
- Phase1: Allows $A_{1}$ to query for polynomially many times as follows.
  1. Key retrieve queries: $C_{1}$ calls KeyGen(i,pp,msk) algorithm and sends SK to $A_{1}$. call the algorithm and send the result to A
  2. Decryption queries: $C_{1}$ runs $Decrypt(pp,CT,\alpha_{i},i)$ algorithm and returns *M*(which might be ⊥) to $A_{1}$.
  3. Authorization queries: For three types of authorization Aut-γ (γ = 1, 2, 3),
    (a) *i* as input, $C_{1}$ sends $TD_{1}$ to $A_{1}$.
    (b) (i,CT) as input, $C_{1}$ sends $TD_{2}$ to $A_{1}$.
    (c) $\left(i,CT,j,CT^{{}^{\prime }}\right)$ as input, $C_{1}$ sends $TD_{3}$ to $A_{1}$.
- Challenge: Adversary $A_{1}$ picks a target identity *t* which has not been queried in extract queries, and sends it to $C_{1}$. Then $C_{1}$ chooses a message $M_{t}$ randomly, gets $C_{t}^{*}=Encrypt\left(M_{t},t,pp\right)$ as the challenge ciphertext and sends it to $A_{1}$.
- Phase2: $A_{1}$ continues issuing the same query as Phase 1. However, *t* can not be queried in this phase and $\left(t,C_{t}^{*}\right)$ can not be queried in a decryption query.
- Guess: $A_{1}$ returns a message $M^{\prime }$, if $M^{\prime }=M_{t}$ means $A_{1}$ wins the game.

We give the advantage definition of $A_{1}$ in the Game I as

$$\begin{array}{c} Adv_{IBEET-FA,A_{1}}^{OW-ID-CCA,Aut-\gamma }\left(k\right)=P\left[M^{\prime }=M_{t}\right]\left(\gamma =1,2,3\right). \end{array}$$

**Definition** **6.** *If the advantage $Adv_{IBEET-FA,A_{1}}^{OW-ID-CCA,Aut-\gamma }\left(k\right)$ is negligible for any probabilistic polynomial-time Adv-I $A_{1}$, We say the IBEET-FA scheme is OW-ID-CCA secure for three types of authorization Aut-γ (γ = 1, 2, 3).*

GameII: Let the recipient’s identity be *t* (1≤t≤n), and Sets $A_{2}$ as an Adv-II adversary. Between the challenger $C_{2}$ and the adversary $A_{2}$ the game goes as follows:

- Setup: Challenger $C_{2}$ firstly picks *k* as a security parameter, then gets public parameter pp by calling Setup algorithm, and sends pp to $A_{2}$.
- Phase1: Allows $A_{2}$ to issue polynomially times queries as in Game I.
- Challenge: Adversary $A_{2}$ sends to Challenger $C_{2}$ two messages $M_{0}$, $M_{1}$, and a target identity *t*, *t* can not be allowed to appear in extract query or Aut-1 authorization query phase. $C_{2}$ picks a bit b∈{0,1} randomly, uses encryption algorithm to get challenge ciphertext $C^{*}=Encrypt\left(M_{b},t,pp\right)$, then sends $C^{*}$ to $A_{2}$.
- Phase2: Allows $A_{2}$ to continue issuing queries as Phase 1, but there are some restrictions as follows:
  1. *i* can not be queried in the Key retrieve query or Aut-1 authorizations queries;
  2. $\left(i,C^{*}\right)$ can not be queried in the decryption query;
  3. $\left(i,C^{*}\right)$ can not be queried in the authorizations query.
- Guess: $A_{2}$ returns a bit $b^{\prime }$, when $b^{\prime }=b$ holds, $A_{2}$ wins in the game.

In Game II, the advantage definition of $A_{2}$ is

$$\begin{array}{c} Adv_{IBEET-FA,A_{2}}^{IND-ID-CCA,Aut-\gamma }\left(k\right)=\left|P\left[b^{\prime }-b\right]-\frac{1}{2}\right|\left(\gamma =1,2,3\right). \end{array}$$

**Definition** **7.** *If the advantage $Adv_{IBEET-FA,A_{2}}^{IND-ID-CCA,Aut-\gamma }\left(k\right)$ is negligible for any probabilistic polynomial-time Adv-II $A_{2}$, We say the IBEET-FA scheme is IND-ID-CCA secure for three types of authorization Aut-γ (γ = 1, 2, 3).*

## 4. Our Proposed IBEET-FA Scheme

In our IBEET-FA scheme, we use the advantages of the PKEET-FA scheme and IBE without pairing scheme.

### 4.1. The Proposed Scheme

- Setup(k): Here *k* is a security parameter, and it is the size of plaintext messages, the algorithm works as follows:
  1. This algorithm calls the TDLGen algorithm of the TDL generator, then gets a tuple (T,G,g,q) where *T* is the trapdoor, *G* is a group, *g* is a random generator, and *q* is the prime order.
  2. Picks some secure hash functions: $H,H_{1}:{\left\{0,1\right\}}^{*}\to G$, $H_{2}:G\to {\left\{0,1\right\}}^{k}$, $H_{3},H_{4}:{\left\{0,1\right\}}^{k}\to Z_{q}$, and $H_{5}:G^{3}\to {Z_{q}}^{2}$.
    Gets the master secret key msk=T, the public parameter $pp=\{H,H_{1},H_{2},H_{3},H_{4},H_{5},G,g,q,k\}$.
- KeyGen(i,pp,msk): Choosing label *i*, the public parameter pp and master secret key msk as input, then calls SolveDL algorithm. H(i) as input, get a value $\alpha_{i}\in Z_{q}$ such that $g^{\alpha_{i}}=H\left(i\right)$. Furthermore, calls SolveDL algorithm again taking $H_{1}\left(i\right)$ as input to get a value $\beta_{i}\in Z_{q}$ such that $g^{\beta_{i}}=H_{1}\left(i\right)$. Then outputs the secret key $sk_{i}=\left(\alpha_{i},\beta_{i}\right)$.
- Encrypt(M,i,pp): Taking a plaintext *M*, public parameter pp and user *i* as input, the algorithm works as follows:
  1. Compute one point $P=(H_{3}\left(M\right),H_{4}\left(M\right))$.
  2. *O* is the origin, use point *P*, *O* to construct a ray f(x) with *O* as the endpoint.
  3. Choose a non zero point $x_{i}\in {\left\{0,1\right\}}^{l}$, then compute $y_{i}=f\left(x_{i}\right)$.
  4. Choose at random $r\in {Z_{q}}^{*}$, then compute
    $$\begin{array}{cc} CT_{1} & =g^{r},\\ CT_{2} & =M⊕H_{2}\left(H{\left(i\right)}^{r}\right),\\ CT_{3} & =\left(x_{i}∥y_{i}\right)⊕H_{5}\left(H_{1}{\left(i\right)}^{r},CT_{1},CT_{2}\right). \end{array}$$
    Output the ciphertext $CT=(CT_{1},CT_{2},CT_{3})$.
- Decrypt(i,CT,SK,pp): Taking label *i*, a ciphertext CT, private key SK and public parameter pp as input, this algorithm computes $M=CT_{2}⊕H_{2}\left(CT_{1}^{\alpha_{i}}\right)$ and $x_{i}∥y_{i}=CT_{3}⊕H_{5}\left(CT_{1}^{\beta_{i}},CT_{1},CT_{2}\right)$. Obtain point *P* as in Encrypt algorithm and obtain f(x) with *P*, *O* as in Encrypt algorithm. if $y_{i}=f\left(x_{i}\right)$ hold, then returns *M*; and returns an error symbol ⊥ otherwise.

Two users are represented as $u_{i}$ and $u_{j}$, selecting $r_{i}$ and $r_{j}$ as the randomness used in computing CT and $CT^{{}^{\prime }}$. Correspondingly, compute ciphertext $CT=(CT_{1},CT_{2},CT_{3})$ and ciphertext $CT^{{}^{\prime }}=(CT_{1}^{{}^{\prime }},CT_{2}^{{}^{\prime }},CT_{3}^{{}^{\prime }}$) of $u_{i}$ and $u_{j}$.

Aut-1:

- $Aut_{1}\left(i,SK\right)$: This authorization procedure returns a trapdoor $TD_{1}=\beta_{i}$.
- $Test_{1}\left(CT,CT^{{}^{\prime }},TD_{1},TD_{1}^{{}^{\prime }}\right)$: The test procedure performs the following calculations
  $$\begin{array}{cc} x_{i}∥y_{i} & =CT_{3}⊕H_{5}\left({CT_{1}}^{TD_{1}},CT_{1},CT_{2}\right),\\ x_{j}∥y_{j} & =CT_{3}^{{}^{\prime }}⊕H_{5}\left({CT_{1}^{{}^{\prime }}}^{TD_{1}^{{}^{\prime }}},CT_{1}^{{}^{\prime }},CT_{2}^{{}^{\prime }}\right). \end{array}$$
  It returns 1 if $\frac{y_{i}}{x_{i}}=\frac{y_{j}}{x_{j}}$, or returns 0 otherwise.

Aut-2:

- $Aut_{2}\left(SK,CT\right)$: This authorization procedure outputs a trapdoor $TD_{2}=H_{5}\left({CT_{1}}^{\beta_{i}},CT_{1},CT_{2}\right)$.
- $Test_{2}\left(CT,CT^{{}^{\prime }},TD_{2},TD_{2}^{{}^{\prime }}\right)$: This test procedure computes
  $$\begin{array}{cc} x_{i}∥y_{i} & =CT_{3}⊕TD_{2},\\ x_{j}∥y_{j} & =CT_{3}^{{}^{\prime }}⊕TD_{2}^{{}^{\prime }}. \end{array}$$
  It returns 1 if $\frac{y_{i}}{x_{i}}=\frac{y_{j}}{x_{j}}$, or returns 0 otherwise.

Aut-3:

- $Aut_{3}\left(SK,CT,CT^{{}^{\prime }}\right)$: This authorization procedure recovers $y_{i}$ with SK, then outputs a trapdoor
  $$\begin{array}{cc} TD_{3} & =(TD_{i,1},TD_{i,2})\\ \; & =({\left[H_{2}\left({CT_{1}}^{\beta_{i}},CT_{1},CT_{2}\right)\right]}_{0}^{l-1},{\left(CT_{1}CT_{1}^{{}^{\prime }}\right)}^{y_{i}}). \end{array}$$
- $Test_{3}\left(CT,CT^{{}^{\prime }},TD_{3},TD_{3}^{{}^{\prime }}\right)$: This test procedure computes
  $$\begin{array}{cc} x_{i} & ={\left[CT_{1}\right]}_{0}^{l-1}⊕TD_{i,1},\\ x_{j} & ={\left[CT_{1}^{{}^{\prime }}\right]}_{0}^{l-1}⊕TD_{j,1}. \end{array}$$
  It returns 1 if ${TD_{i,2}}^{\frac{1}{x_{i}}}={TD_{j,2}}^{\frac{1}{x_{j}}}$, or returns 0 otherwise.

Aut-4:

- $Aut_{4}\left(SK,CT\right)$: This authorization procedure returns a trapdoor $TD_{4}=TD_{2}=H_{5}\left({CT_{1}}^{\beta_{i}},CT_{1},CT_{2}\right)$.
- $Aut_{4}\left(j,SK^{{}^{\prime }}\right)$: This authorization procedure returns a trapdoor $TD_{4}^{{}^{\prime }}=TD_{1}^{{}^{\prime }}=\beta_{j}$.
- $Test_{4}\left(CT,CT^{{}^{\prime }},TD_{4},TD_{4}^{{}^{\prime }}\right)$: This test procedure computes
  $$\begin{array}{cc} x_{i}∥y_{i} & =CT_{3}⊕TD_{4},\\ x_{j}∥y_{j} & =CT_{3}^{{}^{\prime }}⊕H_{5}\left({CT_{1}^{{}^{\prime }}}^{TD_{4}^{{}^{\prime }}},CT_{1}^{{}^{\prime }},CT_{2}^{{}^{\prime }}\right). \end{array}$$
  It returns 1 if $\frac{y_{i}}{x_{i}}=\frac{y_{j}}{x_{j}}$, or returns 0.

### 4.2. Correctness

**Theorem** **1.** *By definition 2, the correctness of the above IBEET-FA scheme is proven.*

**Proof** **of** **Theorem** **1.** We now prove our IBEET-FA scheme meets all correctness requirements.

1. The first requirement is satisfied obviously.
2. According to the second requirement, for any $\left(\alpha_{i},\beta_{i}\right)\leftarrow KeyGen\left(msk,pp,i\right)$, $\left(\alpha_{j},\beta_{j}\right)\leftarrow KeyGen\left(msk,pp,j\right)$, $CT=\left(CT_{1},CT_{2},CT_{3}\right)=Encrypt\left(M_{i},i,pp\right)$, $CT^{{}^{\prime }}=\left(CT_{1}^{{}^{\prime }},CT_{2}^{{}^{\prime }},CT_{3}^{{}^{\prime }}\right)=Encrypt\left(M_{j},j,pp\right)$, all the following equations hold.
  - Aut-1: Given $TD_{1}=\beta_{i}$, $TD_{1}^{{}^{\prime }}=\beta_{j}$, get the following:
    $$\begin{array}{cc} x_{i}∥y_{i} & =CT_{3}⊕H_{5}\left({CT_{1}}^{TD_{1}},CT_{1},CT_{2}\right),\\ x_{j}∥y_{j} & =CT_{3}^{{}^{\prime }}⊕H_{5}\left({CT_{1}^{{}^{\prime }}}^{TD_{1}^{{}^{\prime }}},CT_{1}^{{}^{\prime }},CT_{2}^{{}^{\prime }}\right). \end{array}$$
    Because point $\left(x_{i},y_{i}\right)$ is taken from the ray corresponding to $M_{i}$, point $\left(x_{j},y_{j}\right)$ is taken from the ray corresponding to $M_{j}$, if $M_{i}=M_{j}$ means $\left(x_{i},y_{i}\right)$ and $\left(x_{j},y_{j}\right)$ are taken from the same ray. So $\frac{y_{i}}{x_{i}}=\frac{y_{j}}{x_{j}}$ holds if $M_{i}=M_{j}$.
  - Aut-2: Given
    $$\begin{array}{c} TD_{2}=H_{5}\left({CT_{1}}^{\beta_{i}},CT_{1},CT_{2}\right) \end{array}$$
    and
    $$\begin{array}{c} TD_{2}^{{}^{\prime }}=H_{5}\left({CT_{1}^{{}^{\prime }}}^{\beta_{j}},CT_{1}^{{}^{\prime }},CT_{2}^{{}^{\prime }}\right) \end{array}$$
    get the following:
    $$\begin{array}{cc} x_{i}∥y_{i} & =CT_{3}⊕TD_{2},\\ x_{j}∥y_{j} & =CT_{3}^{{}^{\prime }}⊕TD_{2}^{{}^{\prime }}. \end{array}$$
    Because point $\left(x_{i},y_{i}\right)$ is taken from the ray corresponding to $M_{i}$, point $\left(x_{j},y_{j}\right)$ is taken from the ray corresponding to $M_{j}$, if $M_{i}=M_{j}$ means $\left(x_{i},y_{i}\right)$ and $\left(x_{j},y_{j}\right)$ are taken from the same ray. So $\frac{y_{i}}{x_{i}}=\frac{y_{j}}{x_{j}}$ holds if $M_{i}=M_{j}$.
  - Aut-3: Given
    $$\begin{array}{c} TD_{3}=\left(TD_{i,1},TD_{i,2}\right)=\left({\left[H_{2}\left({CT_{1}}^{\beta_{i}},CT_{1},CT_{2}\right)\right]}_{0}^{l-1},{\left(CT_{1}CT_{1}^{{}^{\prime }}\right)}^{y_{i}}\right) \end{array}$$
    and
    $$\begin{array}{c} TD_{3}^{{}^{\prime }}=\left(TD_{j,1},TD_{j,2}\right)=\left({\left[H_{2}\left({CT_{1}^{{}^{\prime }}}^{\beta_{j}},CT_{1}^{{}^{\prime }},CT_{2}^{{}^{\prime }}\right)\right]}_{0}^{l-1},{\left(CT_{1}^{{}^{\prime }}CT_{1}\right)}^{y_{j}}\right), \end{array}$$
    get the following:
    $$\begin{array}{cc} x_{i} & ={\left[C_{i,3}\right]}_{0}^{l-1}⊕TD_{i,1},\\ x_{j} & ={\left[C_{j,3}\right]}_{0}^{l-1}⊕TD_{j,1},\\ {TD_{i,2}}^{\frac{1}{x_{i}}} & ={\left({\left(C_{i,1}C_{j,1}\right)}^{y_{i}}\right)}^{\frac{1}{x_{i}}}={\left(C_{i,1}C_{j,1}\right)}^{\frac{y_{i}}{x_{i}}},\\ {TD_{j,2}}^{\frac{1}{x_{j}}} & ={\left({\left(C_{j,1}C_{i,1}\right)}^{y_{j}}\right)}^{\frac{1}{x_{j}}}={\left(C_{j,1}C_{i,1}\right)}^{\frac{y_{j}}{x_{j}}}. \end{array}$$
    Because point $\left(x_{i},y_{i}\right)$ is taken from the ray corresponding to $M_{i}$, point $\left(x_{j},y_{j}\right)$ is taken from the ray corresponding to $M_{j}$, if $M_{i}=M_{j}$ means $\left(x_{i},y_{i}\right)$ and $\left(x_{j},y_{j}\right)$ are taken from the same ray. So ${TD_{i,2}}^{\frac{1}{x_{i}}}={TD_{j,2}}^{\frac{1}{x_{j}}}$, i.e., $\frac{y_{i}}{x_{i}}=\frac{y_{j}}{x_{j}}$ holds if $M_{i}=M_{j}$.
  - Aut-4: Given $TD_{4}=TD_{2}=H_{5}\left({CT_{1}}^{\beta_{i}},CT_{1},CT_{2}\right)$, $TD_{4}^{{}^{\prime }}=TD_{1}^{{}^{\prime }}=\beta_{j}$, get the following:
    $$\begin{array}{cc} x_{i}∥y_{i} & =CT_{3}⊕TD_{4},\\ x_{j}∥y_{j} & =CT_{3}^{{}^{\prime }}⊕H_{5}\left({CT_{1}^{{}^{\prime }}}^{TD_{4}^{{}^{\prime }}},CT_{1}^{{}^{\prime }},CT_{2}^{{}^{\prime }}\right). \end{array}$$
    Because point $\left(x_{i},y_{i}\right)$ is taken from the ray corresponding to $M_{i}$, point $\left(x_{j},y_{j}\right)$ is taken from the ray corresponding to $M_{j}$, if $M_{i}=M_{j}$ means $\left(x_{i},y_{i}\right)$ and $\left(x_{j},y_{j}\right)$ are taken from the same ray. So $\frac{y_{i}}{x_{i}}=\frac{y_{j}}{x_{j}}$ holds if $M_{i}=M_{j}$.
3. Now we prove the third condition holds.
  $F_{i}\left(x\right)$ is a ray passing through point $P_{i}=\left(H_{3}\left(M_{i}\right),H_{4}\left(M_{i}\right)\right)$ with *O* as its endpoint, $f_{j}\left(x\right)$ is a ray passing through $P_{j}=\left(H_{3}\left(M_{j}\right),H_{4}\left(M_{j}\right)\right)$ with *O* as its endpoint. Point $\left(x_{i},y_{i}\right)$ is taken from the ray $f_{i}\left(x\right)$, and point $\left(x_{j},y_{j}\right)$ is taken from the ray $f_{j}\left(x\right)$.
  - Aut-1: If $Test_{1}\left(CT,CT^{{}^{\prime }},TD_{1},TD_{1}^{{}^{\prime }}\right)=1$, we can get that $\frac{y_{i}}{x_{i}}=\frac{y_{j}}{x_{j}}$, that is, point $\left(x_{i},y_{i}\right)$ and point $\left(x_{j},y_{j}\right)$ are taken from the same ray with *O* as the end point. For $M_{i}\neq M_{j}$, $P[\frac{H_{4}\left(M_{i}\right)}{H_{3}\left(M_{i}\right)}=\frac{H_{4}\left(M_{j}\right)}{H_{3}\left(M_{j}\right)}]$ is negligible, then we get that $P[Test_{1}\left(CT,CT^{{}^{\prime }},TD_{1},TD_{1}^{{}^{\prime }}\right)=1]$ is also negligible for $M_{i}\neq M_{j}$.
  - Aut-2: If $Test_{2}\left(CT,CT^{{}^{\prime }},TD_{2},TD_{2}^{{}^{\prime }}\right)=1$, we can get that $\frac{y_{i}}{x_{i}}=\frac{y_{j}}{x_{j}}$, that is, point $\left(x_{i},y_{i}\right)$ and point $\left(x_{j},y_{j}\right)$ are taken from the same ray with *O* as the end point. For $M_{i}\neq M_{j}$, $P[\frac{H_{4}\left(M_{i}\right)}{H_{3}\left(M_{i}\right)}=\frac{H_{4}\left(M_{j}\right)}{H_{3}\left(M_{j}\right)}]$ is negligible, then we get that $P[Test_{2}\left(CT,CT^{{}^{\prime }},TD_{2},TD_{2}^{{}^{\prime }}\right)=1]$ is also negligible.
  - Aut-3: If $Test_{3}\left(CT,CT^{{}^{\prime }},TD_{3},TD_{3}^{{}^{\prime }}\right)=1$, we can get that ${TD_{i,2}}^{\frac{1}{x_{i}}}={TD_{j,2}}^{\frac{1}{x_{j}}}$, that is, ${\left(C_{i,1}C_{j,1}\right)}^{\frac{y_{i}}{x_{i}}}={\left(C_{j,1}C_{i,1}\right)}^{\frac{y_{j}}{x_{j}}}$. For $M_{i}\neq M_{j}$, $P[\frac{H_{4}\left(M_{i}\right)}{H_{3}\left(M_{i}\right)}=\frac{H_{4}\left(M_{j}\right)}{H_{3}\left(M_{j}\right)}]$ is negligible, we get that $P[Test_{3}\left(CT,CT^{{}^{\prime }},TD_{3},TD_{3}^{{}^{\prime }}\right)=1]$ is also negligible for $M_{i}\neq M_{j}$.
  - Aut-4: If $Test_{4}\left(CT,CT^{{}^{\prime }},TD_{4},TD_{4}^{{}^{\prime }}\right)=1$, we can get that $\frac{y_{i}}{x_{i}}=\frac{y_{j}}{x_{j}}$, that is, point $\left(x_{i},y_{i}\right)$ and point $\left(x_{j},y_{j}\right)$ are taken from the same ray with *O* as the end point. For $M_{i}\neq M_{j}$, $P[\frac{H_{4}\left(M_{i}\right)}{H_{3}\left(M_{i}\right)}=\frac{H_{4}\left(M_{j}\right)}{H_{3}\left(M_{j}\right)}]$ is negligible, we get that $P[Test_{4}\left(CT,CT^{{}^{\prime }},TD_{4},TD_{4}^{{}^{\prime }}\right)=1]$ is also negligible for $M_{i}\neq M_{j}$.

□

## 5. Security Analysis

We will prove two kinds of security against different adversaries in this section. For this purpose, we design several related games to connect the scheme security and the hardness problems. Suppose A is a polynomial-time adversary, allowing A to do at most $q_{H}$, $q_{H_{1}}$, $q_{H_{2}}$, $q_{H_{3}}$, $q_{H_{4}}$, $q_{H_{5}}$ times of queries to hash oracles $O_{H}$, $O_{H_{1}}$, $O_{H_{2}}$, $O_{H_{3}}$, $O_{H_{4}}$, $O_{H_{5}}$,respectively, $q_{K}$ times key generation queries, $q_{D}$ times decryption queries, $q_{T}$ times trapdoor queries. Challenger C controls oracles and answers the queries of adversaries. $L_{H}$, $L_{H_{1}}$, $L_{H_{2}}$, $L_{H_{3}}$, $L_{H_{4}}$, $L_{H_{5}}$ stand for hash lists.

### 5.1. OW-ID-CCA Security Against Adv-I

**Theorem** **2.** *Based on CDH assumption, in the random oracle model our presented IBEET-FA scheme is OW-ID-CCA secure against Adv-I for Aut-γ (γ = 1, 2, 3) authorization.*

**Proof** **of** **Theorem** **2.** We design several related games to prove OW-ID-CCA security against Adv-I $A_{1}$. Let P[$Game_{i}$] present the probability of breaking game *i*, where i∈{1,2,3}.

*Game*1:  

- Setup: The challenger $C_{1}$ outputs public parameter {G,g,q,k}, the master secret key msk=T.
- Phase1: Allows $A_{1}$ to do the following queries.
  1. Hash queries: Suppose $A_{1}$ queries at most $q_{H}$, $q_{H_{1}}$, $q_{H_{2}}$, $q_{H_{3}}$, $q_{H_{4}}$, $q_{H_{5}}$ times to hash oracles $O_{H}$, $O_{H_{1}}$, $O_{H_{2}}$, $O_{H_{3}}$, $O_{H_{4}}$, $O_{H_{5}}$, respectively.
    (a) $O_{H}$, $O_{H_{1}}$: Set original empty lists $L_{H}$(resp.$L_{H_{1}}$). For an identity *i*, the oracle picks $r_{i_{1}}\leftarrow Z_{q}$(resp.$r_{i_{2}}\leftarrow Z_{q}$) randomly, computes $H\left(i\right)=g^{r_{i_{1}}}$(resp.$H_{1}\left(i\right)=g^{r_{i_{2}}}$) and records the tuple $\left(i,r_{i_{1}},g^{r_{i_{1}}}\right)$(resp.$\left(i,r_{i_{2}},g^{r_{i_{2}}}\right)$) on hash list $L_{H}$(resp.$L_{H_{1}}$). H(i)(resp.$H_{1}\left(i\right)$) is returned to $A_{1}$.
    (b) $O_{H_{2}}$: Set original empty lists $L_{H_{2}}$. For an input $U_{i}$, the oracle picks a string $S_{i}\in {\left\{0,1\right\}}^{k}$ randomly and records the tuple $\left(U_{i},S_{i}\right)$ on hash list $L_{H_{2}}$. $H_{2}\left(U_{i}\right)=S_{i}$ is returned to $A_{1}$.
    (c) $O_{H_{3}}$, $O_{H_{4}}$: Set original empty lists $L_{H_{3}}$. For an input $S_{i}$, the oracle picks $r_{i}\leftarrow Z_{q}$ randomly and records the tuple $\left(S_{i},r_{i}\right)$ on hash list $L_{H_{3}}$. $H_{3}\left(S_{i}\right)=r_{i}$ is returned to $A_{1}$.
    (d) $O_{H_{5}}$: Set original empty lists $L_{H_{5}}$. For an input $U_{i}$, the oracle picks a string $S_{i}\in {\left\{0,1\right\}}^{2l}$ randomly and records the tuple $\left(U_{i},S_{i}\right)$ on hash list $L_{H_{5}}$. $H_{5}\left(U_{i}\right)=S_{i}$ is returned to $A_{1}$.
  2. Key retrieve queries: For an identity *i*, challenger $C_{1}$ invokes hash oracles $O_{H}$, $O_{H_{1}}$ to get hash values H(i), $H_{1}\left(i\right)$, then runs KeyGen(msk,pp,i) algorithm to get the secret key $sk_{i}=\left(\alpha_{i},\beta_{i}\right)$. It returns $sk_{i}$ to $A_{1}$.
  3. Decryption queries: For an identity *i*, ciphertext $C_{i}$, challenger $C_{1}$ invokes key retrieve queries to obtain the secret key $sk_{i}=\left(\alpha_{i},\beta_{i}\right)$, then uses $sk_{i}$ to call $Decrypt(pp,C_{i},\alpha_{i},i)$ algorithm to obtain the message $M_{i}$(which might be ⊥). It returns $M_{i}$ (or ⊥) to $A_{1}$.
  4. Authorization queries: For Aut-γ (γ = 1, 2, 3),
    (a) γ=1: *i* as the input, $C_{1}$ runs $Aut_{1}$ algorithm with SK, then returns $TD_{1}=\beta_{i}$ to $A_{1}$.
    (b) γ=2: (i,CT) as the input, $C_{1}$ runs $Aut_{2}$ algorithm with SK, then returns $TD_{2}=H_{5}\left({CT_{1}}^{\beta_{i}},CT_{1},CT_{2}\right)$ to $A_{1}$.
    (c) γ=3: $\left(i,CT,j,CT^{{}^{\prime }}\right)$ as the input, $C_{1}$ runs $Aut_{3}$ algorithm with SK, then returns $TD_{3}=\left({\left[H_{2}\left({CT_{1}}^{\beta_{i}},CT_{1},CT_{2}\right)\right]}_{0}^{l-1},{\left(CT_{1}CT_{1}^{{}^{\prime }}\right)}^{y_{i}}\right)$ to $A_{1}$.
- Challenge: Adversary $A_{1}$ submits to $C_{1}$ an identity *t*, and *t* has not been queried in previous extract query, $C_{1}$ randomly selects a message $M_{t}$, and gets $C_{t}^{*}=\left(C_{t,1}^{*},C_{t,2}^{*},C_{t,3}^{*}\right)$ with the following equations.
  $$\begin{array}{cc} C_{t,1}^{*} & =g^{r_{t}},\\ C_{t,2}^{*} & =M_{t}⊕H_{2}\left(H{\left(t\right)}^{r_{t}}\right),\\ C_{t,3}^{*} & =\left(x_{t}∥y_{t}\right)⊕H_{5}\left(H_{1}{\left(t\right)}^{r_{t}},C_{t,1}^{*},C_{t,2}^{*}\right), \end{array}$$
  where the point $\left(x_{t},y_{t}\right)$ is randomly taken from the ray passing through the point $\left(H_{3}\left(M_{t}\right),H_{4}\left(M_{t}\right)\right)$, and $r_{t}\in {Z_{q}}^{*}$. Then, the challenge ciphertext $C_{t}^{*}$ is sent to $A_{1}$.
- Phase2: Allows $A_{1}$ to issue the same type query as in Phase 1. However, in the key retrieve queries, *t* can not be allowed to query; and in the decryption queries, $\left(t,C_{t}^{*}\right)$ can not be queried.
- Guess: $A_{1}$ returns a message $M^{\prime }$, if $M^{\prime }=M_{t}$, means in the game $A_{1}$ wins. The probability of adversary $A_{1}$ winning the game is:
  $$\begin{array}{c} Adv_{IBEET-FA,A_{1}}^{OW-ID-CCA,Aut-\gamma }\left(k\right)=P\left[Game_{1}\right]\left(\gamma =1,2,3\right). \end{array}$$

*Game*2:  It is almost equivalent to Game 1, the modified parts are as follows:

$$\begin{array}{cc} C_{i,1} & =g^{r},\\ C_{i,2} & =M⊕R,\\ C_{i,3} & =\left(x_{i}∥y_{i}\right)⊕H_{5}\left(H_{1}{\left(i\right)}^{r},C_{i,1}^{*},C_{i,2}^{*}\right). \end{array}$$

The change is that $H_{2}\left(H{\left(t\right)}^{r_{t}}\right)$ is replaced by a random *R*. We can see that $H_{2}\left(H{\left(t\right)}^{r_{t}}\right)$ is random in Game1. If $H{\left(t\right)}^{r_{t}}$ has been queried in Game2, we call it event *E*. If $H{\left(t\right)}^{r_{t}}$ has not been queried, it is difficult for $A_{1}$ to separate Game1 and Game2. We get that

$$\begin{array}{c} |P[Game1]-P[Game2]|\leq P[E], \end{array}$$

then have

$$\begin{array}{c} P[Game1]\leq P[Game2]+P[E]. \end{array}$$

Obviously, P[E] is ignorable if the CDH problem is difficult.

*Game*3:  It is almost equivalent to *Game*2, the modified parts are as follows:

$$\begin{array}{cc} C_{i,1} & =g^{r},\\ C_{i,2} & =R_{1},\\ C_{i,3} & =\left(x_{i}∥y_{i}\right)⊕H_{5}\left(H_{1}{\left(i\right)}^{r},C_{i,1}^{*},C_{i,2}^{*}\right). \end{array}$$

Compared to Game2, M⊕R in Game3 is changed by random $R_{1}$. *R* is a random string, we can konw that M⊕R is also a random string. So it is difficult for $A_{1}$ to separate Game2 and Game3. We have that

$$\begin{array}{c} P[Game2]=P[Game3] \end{array}$$

Similarly, if CDH problem is difficult, P[Game3] is ignorable.

From all the formulas obtained above, we derive the following formula

$$\begin{array}{c} P[Game1]\leq P[Game2]+P[E]\leq P[Game3]+P[E] \end{array}$$

We can get a conclusion: when the CDH problem is intractable, our new IBEET-FA scheme can achieve IND-ID-CCA security against Adv-I. □

### 5.2. IND-ID-CCA Security Against Adv-II

**Theorem** **3.** *Based on DDH assumption, in the random oracle model our presented IBEET-FA scheme is IND-ID-CCA secure against Adv-II for Aut-γ (γ = 1, 2, 3) authorization.*

**Proof**  (Proof of Theorem 3). If such an adversary $A_{2}$ exists who could attack the IND-ID-CCA security of this scheme, we then can get an algorithm to solve the DDH problem in polynomial time with not negligible advantage. For Adv-II $A_{2}$, we design the following game to prove the IND-ID-CCA security. The probability of winning the game is expressed as P[Game].

For $a,b,c\in Z_{q}$, given two tuples $\left(g,g^{a},g^{b},g^{ab}\right),\left(g,g^{a},g^{b},g^{c}\right)\in G$, $C_{2}$ computes system parameters and sends to $A_{2}$. For the queries of $A_{2}$, $C_{2}$ replies as following.

- Setup: For i∈[1,n], algorithm $C_{2}$ generates *n* key pairs $\left(sk_{i},pk_{i}\right)$, where sets $\left(sk_{i},pk_{i}\right)=\left(\left(\alpha_{i},\beta_{i}\right),\left(g^{\alpha_{i}},g^{\beta_{i}}\right)\right)\left(\alpha_{i},\beta_{i}\in Z_{q}\right)$.
- Phase1: Allows algorithm $C_{2}$ to issue four types of queries as follows.
  1. Hash queries:
    (a) $O_{H}$, $O_{H_{1}}$: Work in the same way as in Game1.
    (b) $O_{H_{2}}$: Works in the same way as in Game1.
    (c) $O_{H_{3}}$, $O_{H_{4}}$: Works in the same way as in Game1.
    (d) $O_{H_{5}}$: Works in the same way as in Game1.
  2. Key retrieve queries: Given an identity *i*, $C_{2}$ searches tuple $\left(i,r_{i_{1}},g^{r_{i_{1}}}\right)$ and tuple $\left(i,r_{i_{2}},g^{r_{i_{2}}}\right)$ in list $L_{H}$ and list $L_{H_{1}}$, sends $\left(r_{i_{1}},r_{i_{2}}\right)$ to $A_{2}$ when i≠t holds. Otherwise, $C_{2}$ returns ⊥ to $A_{2}$.
  3. Decryption queries: For identity *i* and a query ciphertext $C_{i}$, challenger $C_{2}$ searches tuple (U,S) in list $L_{H_{2}}$, and computes $M\|R=C_{2}+S$. If exists *R*, making equation $C_{1}=g^{R}$ true, $C_{2}$ returns *M* to $A_{2}$. Otherwise, $C_{2}$ returns ⊥ to $A_{2}$.
  4. Authorization queries: For Aut-γ (γ = 1, 2, 3),
    (a) γ=1: *i* as the input, challenger $C_{2}$ calls $Aut_{1}$ algorithm with SK, then sends $TD_{1}=\beta_{i}$ to $A_{2}$.
    (b) γ=2: (i,CT) as the input, challenger $C_{2}$ calls $Aut_{2}$ algorithm with SK, then sends $TD_{2}=H_{5}\left({CT_{1}}^{\beta_{i}},CT_{1},CT_{2}\right)$ to $A_{2}$.
    (c) γ=3: given $\left(i,CT,j,CT^{{}^{\prime }}\right)$ as input, challenger $C_{2}$ calls $Aut_{3}$ algorithm with SK, then sends $TD_{3}=\left({\left[H_{2}\left({CT_{1}}^{\beta_{i}},CT_{1},CT_{2}\right)\right]}_{0}^{l-1},{\left(CT_{1}CT_{1}^{{}^{\prime }}\right)}^{y_{i}}\right)$ to $A_{2}$.
- Challenge: Adversary $A_{2}$ chooses two plaintext $M_{0}$, $M_{1}$ and an identity *t*, there is a contraint that *t* can not be queried in extract queriy phase or Aut-1 authorization query phase. $C_{2}$ picks a bit b∈{0,1} randomly, then encrypts $M_{b}$:
  $$\begin{array}{cc} C_{t,1}^{*} & =g^{x},\\ C_{t,2}^{*} & =M_{b}⊕H_{2}\left(g^{z}\right),\\ C_{t,3}^{*} & =\left(x_{t}∥y_{t}\right)⊕H_{5}\left(H_{1}{\left(t\right)}^{r_{t}},C_{t,1}^{*},C_{t,2}^{*}\right), \end{array}$$
  challenger $C_{2}$ sends the obtained challenge ciphertext $C^{*}=\left(C_{t,1}^{*},C_{t,2}^{*},C_{t,3}^{*}\right)$ to the adversary $A_{2}$.
- Phase2: $A_{2}$ issues the same type query as in Phase 1, and there are two following restrictions:
  1. In the key retrieve query phase or Aut-1 authorizations query phase, *i* could not be allowed to query;
  2. In the decryption query phase or the authorization query phase, $\left(i,C^{*}\right)$ could not be queried.
- Guess: $A_{2}$ returns a bit $b^{\prime }$. If $b^{\prime }=b$ holds, it means that $A_{2}$ wins the game, then $C_{2}$ outputs 1.

□

## 6. Efficiency Analysis

In Table 1, we describe the communication complexity of our scheme, and compare it with other schemes [4,5,23,24]. $|Z_{p}|$, |G|, $|G_{1}|$ and $|G_{T}|$ are used to represent the size of elements in $Z_{p}$, *G*, $G_{1}$ and $G_{T}$, the second column represents the size of the public key, the third column represents the size of a private key, the four columns represent the size of ciphertext. We can see that our scheme has a smaller size than [4,23,24] in public key and ciphertext, and has a smaller size than [5] in the ciphertext.

**Figure 2:**
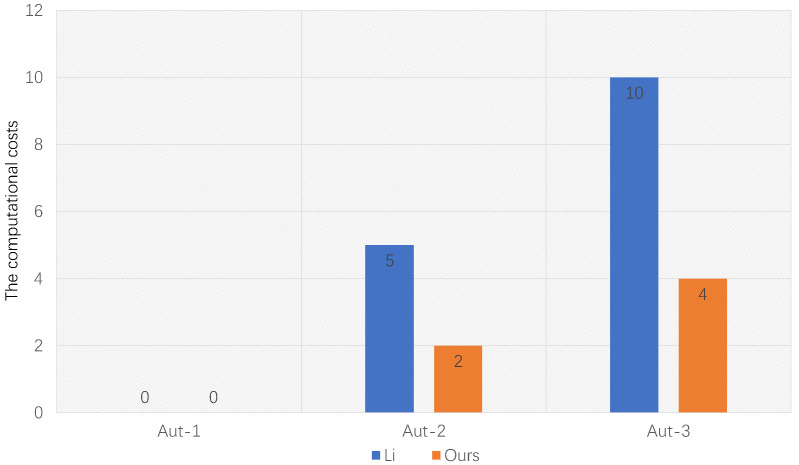
Computational costs comparison of three authorizations with Li [5].

In Table 2, we show the comparison of encryption, decryption, authorization, and test in computation complexity. We use “I”, “E”, and “P” to represent the inversion operation, exponentiation operation and pairing operation, respectively, and represent the comparison of the encryption process, decryption process, authorization process, and test process in computation complexity from the second to fifth columns. In the sixth column, we represent whether the scheme is identity-based, and represents whether the scheme is pairing-based in the last column. Our scheme and [5] have four authorization algorithms. Since Aut-4 is a combination of Aut-1 and Aut-2, we omit Aut-4 for simplicity. In Table 2 and Figure 2, we list the three authorization algorithms of our scheme and [5] for comparison. In the encryption algorithm, Ref. [5] requires seven exponential operations, while our scheme only requires three exponential operations. In the Aut-2 authorization algorithm, Ref. [5] requires one pairing operation, and our scheme only requires two exponential operations. In Aut-3 authorization algorithm, Ref. [5] requires two pairing operations, and our scheme only requires four exponential operations. For the two authorization processes, our scheme reduces the computation costs by 60%, respectively. Reducing the use of pairings is key to reducing computational costs. Compared with [4,23,24], our scheme and [5] are based on identity encryption. The user’s public key can be a string related to the user’s identity information, which avoids complicated public key certificate management and public key storage. However, Refs. [4,23,24] use public key encryption, which requires a large amount of storage and complex management. Among all the schemes we list, our scheme is the only one that can achieve both ID-based and no pairing.

From the comparison results in Figure 2, it can be seen that the calculation costs of the authorization algorithms of the three authorization methods in our scheme are significantly lower than that of the corresponding three authorization algorithms in Li et al.’s scheme [5]. Compared with other schemes [4,5,23,24], our scheme is more flexible and efficient. In cloud computing, our scheme is applicable to more application scenarios and has high practical significance.

## 7. Conclusions

In this paper, we propose a new IBE scheme without pairing, which supports the ciphertext equality test. Our scheme introduces the authorization mechanism proposed in the scheme [4], four types of authorization policies providing better flexibility. Compared with works [4,23,24], our scheme is in IBE settings, which means do not need to suffer from complex key store and distribution problems. Compared with works [5], we replaced pairing with discrete logarithms, which helps reduce the computation cost. Specifically, compared to Li et al.’s work, about 57% = (100%−43%) time cost is saved for the encryption process, and about 60% = (100%−40%) time costs are saved for the type-2 authorization process and type-3 authorization process. Based on mathematical assumptions, we define the security models of our scheme and prove the security of the scheme.

Our proposed approach can be applied to equality tests over ciphertexts encrypted with different public keys, which increases the application range of cloud computing. Furthermore, our scheme is in IBE settings, avoiding complex key management issues. However, there are security channel key distribution and private key escrow issues in IBE. In the future, we will try to combine the advantages of IBE and PKE to propose more secure and efficient equality test schemes.

## Figures and Tables

**Table 1:** Communication complexity.

	Public Key	Secret Key	Ciphertext
PKEET-FA [4]	3|G|	$3|Z_{p}|$	$5|G|+|Z_{p}|$
PKEET-FA [23]	2|G|	$2|Z_{p}|$	$2|G|+6|Z_{p}|$
PKEET-FA [24]	2|G|	$2|Z_{p}|$	$\left|G|+5\right|Z_{p}|$
IBEET-FA [5]	$|G_{1}|$	$2|G_{1}|$	$5|G_{1}\left|+\right|G_{T}\left|+2\right|Z_{p}|$
Our IBEET-FA	$|G_{1}|$	$2|G_{1}|$	$|G_{1}\left|+3\right|Z_{p}|$

**Table 2:** Computation complexity.

	Encryption	Decryption	Authorization	Test	ID-Based	Pairings-Based
PKEET-FA [4] Aut-1	6E	5E	0	2P + 2E	NO	YES
PKEET-FA [4] Aut-2	6E	5E	2E	2P + 2E	NO	YES
PKEET-FA [4] Aut-3	6E	5E	2P + 2E	2P + 2E	NO	YES
PKEET-FA [23] Aut-1	4E + 6I	3E + 6I	0	2E + 6I	NO	NO
PKEET-FA [23] Aut-3	4E + 6I	3E + 6I	4E	6E + 6I	NO	NO
PKEET-FA [24] Aut-1	3E + I	2E + I	0	2E + 2I	NO	NO
PKEET-FA [24] Aut-2	3E + I	2E + I	E	2I	NO	NO
PKEET-FA [24] Aut-3	3E + I	2E + I	3E	4E + 4I	NO	NO
IBEET-FA [5] Aut-1	7E	3P + 2E	0	4P	YES	YES
IBEET-FA [5] Aut-2	7E	3P + 2E	P	2P	YES	YES
IBEET-FA [5] Aut-3	7E	3P + 2E	2P	2P	YES	YES
Our IBEET-FA Aut-1	3E	2E	0	2E + 2I	YES	NO
Our IBEET-FA Aut-2	3E	2E	2E	2I	YES	NO
Our IBEET-FA Aut-3	3E	2E	4E	2E + 2I	YES	NO

## Data Availability

Not applicable.
